# Posterior Vitreous Detachment in Healthy Versus AMD Eyes Assessed by Widefield Optical Coherence Tomography

**DOI:** 10.3390/diagnostics15111382

**Published:** 2025-05-29

**Authors:** Maciej Gawęcki, Krzysztof Kiciński, Andrzej Grzybowski, Sławomir Teper

**Affiliations:** 1 Department of Ophthalmology of Pomeranian Hospitals, 84-120 Wejherowo, Poland; krzysztofkg999@icloud.com; 2 Dobry Wzrok Ophthalmological Clinic, 80-822 Gdansk, Poland; 3 Department of Ophthalmology, University of Warmia and Mazury, Oczapowskiego 2, 10-719 Olsztyn, Poland; ae.grzybowski@gmail.com; 4 Institute for Research in Ophthalmology, Foundation for Ophthalmology Development, Mickiewicza 24, 61-836 Poznan, Poland; 5 Chair and Clinical Department of Ophthalmology, Faculty of Medical Sciences in Zabrze, Medical University of Silesia, 40-752 Katowice, Poland; slawomir.teper@gmail.com

**Keywords:** posterior vitreous detachment, ultrawide field OCT, retinal thickness, AMD

## Abstract

**Introduction:** This study aimed to determine the frequency of posterior vitreous detachment (PVD) in dry and wet age-related macular degeneration (AMD) patients compared with healthy eyes via ultrawide field optical coherence tomography (UWF–OCT). Additionally, the retinal thicknesses in the central and peripheral zones of AMD patients and the control group were compared. **Methods:** We included 123 eyes from 83 participants with dry AMD, 123 from 87 participants with wet AMD, and 85 from 53 healthy controls. All three study groups were compared according to age, sex, best corrected visual acuity (BCVA), PVD stage, axial length, and retinal thickness in the central, perifoveal, and peripheral zones. Additional analyses included correlations between the BCVA and PVD stage and between retinal thickness and the PVD stage. **Results:** Complete separation of the vitreous from the macula was significantly more common in AMD patients than in the control group, as noted in 47 eyes (55.29%) in the control group, 92 eyes (74.80%) in the wet AMD group, and 93 eyes (75.61%) in the dry AMD group. The PVD stage did not significantly influence retinal thickness. BCVA in AMD patients did not correlate with the PVD stage. **Conclusions:** Complete PVD is more common in AMD patients than in healthy controls, as evaluated by UWF–OCT. No relationship between the PVD stage and AMD type or BCVA was observed.

## 1. Introduction

Ultrawide field optical coherence tomography (UWF–OCT) devices have recently been introduced in clinical practice. UWF-OCT allows a large scanning field exceeding 20 mm in length, providing important information on the involvement of the peripheral retina in diseases [1,2]. In particular, UWF–OCT devices provide a distinct view of the peripheral vitreoretinal interface; thus, they are reliable for assessing vitreous detachment or peripheral traction [3,4,5]. Moreover, swept-source (SS) UWF–OCT permits precise measurements of retinal thicknesses at the central and peripheral sectors, enabling correlations between retinal architecture and the condition of the vitreoretinal interface. Few papers evaluating the relationship between the condition of vitreoretinal interface and the retina have been published, especially concerning age-related macular degeneration (AMD). The role of posterior vitreous adhesion in the evolution of AMD has been explored in numerous studies; however, the use of UWF–OCT technology has not been explored.

In this study, we assessed the frequency of posterior vitreous detachment (PVD) determined by UWF–OCT in dry and wet AMD patient eyes versus control group eyes. Additionally, we analyzed the relationship between retinal thickness and PVD stage in the central and peripheral zones.

## 2. Materials and Methods

This study was conducted in accordance with the tenets of the Declaration of Helsinki and approved by the local ethical board of Okręgowa Izba Lekarska no KB-40/24. Study procedures were performed in the Department of Ophthalmology of Specialist Hospital in Chojnice between April and December 2023. The material included 123 eyes from 83 participants with dry AMD, 123 from 87 participants with wet AMD, and 85 from 53 controls. All AMD patients were consecutive and included in the Polish National AMD Treatment Program. Wet AMD patients in this program were treated with intravitreal anti-VEGF injections in accordance with the recommendations for the specific medication applied. The diagnosis was determined according to a series of tests, including a basic ophthalmological examination (best corrected visual acuity (BCVA), anterior and posterior segment slit lamp evaluation, and intraocular pressure measurement), fluorescein angiography, and spectral domain optical coherence tomography (SD–OCT). OCT angiography was performed in selected cases as an auxiliary procedure to determine the exudative form of AMD. Patients with significant opacity of ophthalmic media precluding UWF–OCT scan acquisition were excluded. Additionally, eyes with other ophthalmic comorbidities, including a history of posterior segment ocular inflammation, epiretinal membranes, macular neovascularization due to other diseases, vascular diseases of the retina (diabetic retinopathy, retinal vein occlusion, or retinal artery occlusions), and a history of vitreoretinal surgery, were excluded.

The control group included patients aged 50 years or older who underwent routine screening at the hospital’s outpatient clinic. Only individuals without AMD or other ophthalmic diseases were included in the control group. Those with opacity of the ophthalmic media or amblyopic eyes were excluded.

**Figure 1:**
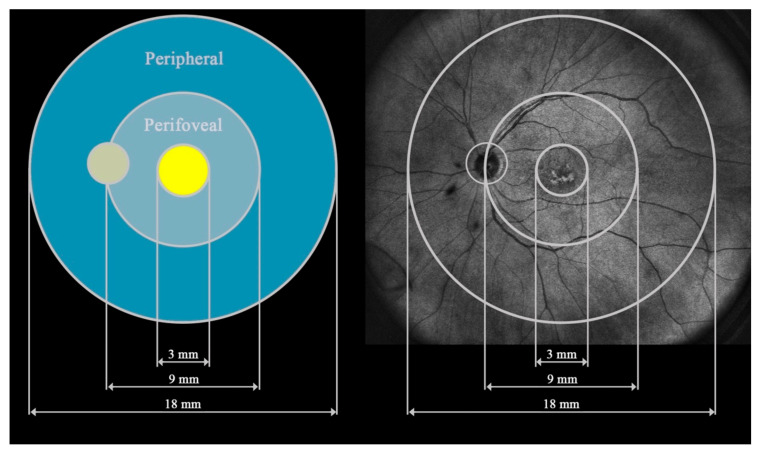
Presentation of the three fields analyzed in this study: central, perifoveal, and peripheral.

All study participants underwent UWF–OCT testing after pupil dilation with a new swept-source device: Xephilio OCT-S1 (Canon Medical Systems Europe B.V., Amstelveen, Netherlands, 2023). The equipment enables the acquisition of scans of 20 × 23 mm without the need for a montage. Retinal thickness (RT) measurements were performed in 24 fields according to the machine’s protocols. For this study, the 24 fields were merged into 3 zones extending from the center to the periphery: the central circle, 3 mm in diameter (central), the ring between the central 9 mm circle and the central 3 mm circle (perifoveal), and the second more peripheral ring between the central 18 mm and 9 mm circles (peripheral) (Figure 1).

The PVD stage was determined according to the obtained UWF scans and classified according to the widely accepted classification proposed by Tsukhahara et al. [6]. Stage 1 refers to early PVD characterized by the subtle separation of the peripheral vitreous cortex visible on OCT scans as a hyperreflective granular zone. In addition to stage 1a signs, stage 1b signs include more apparent separation of the peripheral vitreous cortex from the retina. In stage 2, PVD is clear at the periphery but does not include the fovea. Stage 2 PVD was recognized when the peripheral vitreous cortex was separated closer than 750 (μm) to the foveal center. In stage 3, PVD encompasses almost the entire vitreous, with its attachment still present at the papilla. Stage 4 is characterized by the separation of the vitreous at every point in the retina and optic nerve.

**Figure 2:**
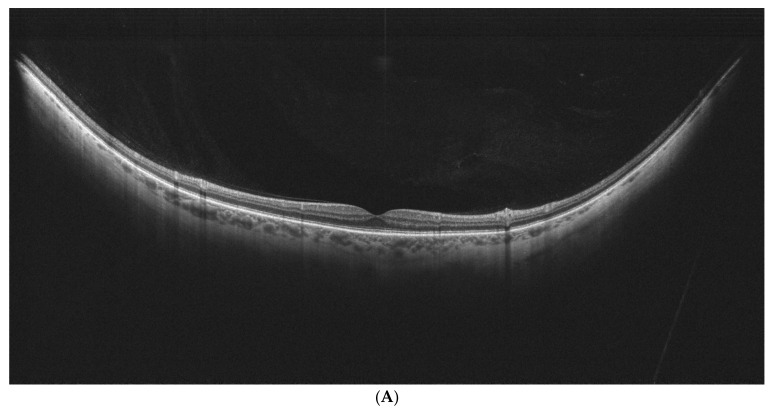

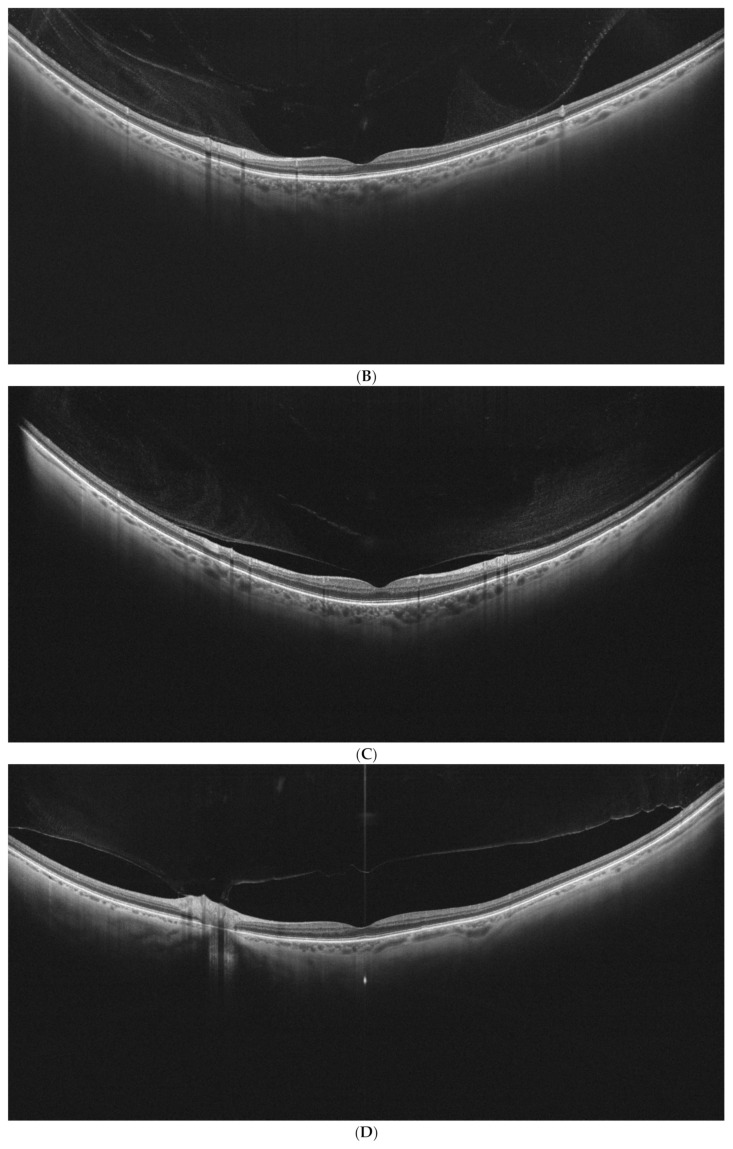

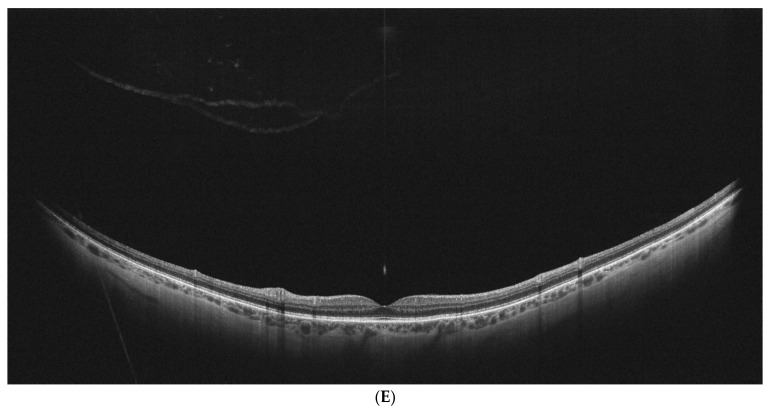
(**A**–**E**). PVD stages as determined by UWF–OCT testing. (**A**) PVD Stage 1a. Hyper-reflective material is present at the border of the vitreoretinal interface, with PVD emerging at the periphery. (**B**) PVD Stage 1 B. Clear separation of the vitreous is visible at the retinal periphery on one side, with the other side still attached. (**C**) PVD Stage 2. Partial vitreous separation is noted in the posterior pole, with a small attachment remaining in the foveal center. (**D**) PVD Stage 3. Complete vitreous separation is noted in the posterior pole, with its cortex still attached at the papilla. (**E**) PVD Stage 4. Complete vitreous separation visible on the UWF–OCT scan.

Examples of stages of PVD on UWF–OCT testing are presented in Figure 2.

AMD stages were determined according to the accepted classification by Ferris et al. [7] after basic fundus examination, color fundus photography, and fluorescein angiography analysis.

All three study groups were compared according to age, sex, BCVA, PVD stage, axial length, and RT in the central, perifoveal, and peripheral zones. Correlations between the PVD stage and BCVA and retinal thickness were also analyzed.

## 3. Statistical Analysis

Categorical variables are shown as integer numbers and percentages (frequencies). Numerical traits are depicted as means, medians, standard deviations, and lower-to-upper quartile values. The normality of distribution was assessed via the Shapiro–Wilk W test. Levene’s test was used to assess the homogeneity of variances. A multifactor analysis of variance (ANOVA) was performed to test the significance of differences in normally distributed numerical traits between the study groups. Generalized linear models (GLMs) were fitted when assessing non-normally distributed numerical variables. Error correction based on intrasubject correlations was applied, as necessitated by the bilateral eye examinations performed. All the multivariate tests mentioned were controlled for the participants’ age and sex. This procedure is indicated in the table subscripts. The rationale behind this approach was the specific etiopathogenesis of the disorder under study and the study participants’ general population-derived diverse age structure. Spearman’s rank correlation coefficients were computed when appraising relationships between selected numerical traits. A level of *p* < 0.05 was considered to indicate significance. All the procedures were performed via Statistica™, release 13.3 (TIBCO Software Inc., Palo Alto, CA, USA).

## 4. Results

The demographic baseline characteristics of the study cohort are provided in Table 1.

The participants in both AMD groups were similar in age and slightly older than those in the control group; hence, all the performed analyses were controlled for age. Female participants were more common in the AMD subgroup; however, a significant difference from the controls was noted only for dry AMD (*p* = 0.0194). Compared with the control group, AMD patients had poorer BCVA, and wet AMD eyes had lower BCVA than dry AMD eyes did. There were no differences in axial length between the study groups. Significant differences in the frequency of different PVD stages between the groups were observed (Table 2 (parts A and B)).

A complete separation of the vitreous from the macula (stages 3 and 4) was more common in AMD patients than in the control group. Stage 3 or 4 disease was noted in 47 eyes (55.29%) in the control group, 92 eyes (74.80%) in the wet AMD group, and 93 eyes (75.61%) in the dry AMD group. Significant differences were detected between the control and wet AMD groups (*p* = 0.0037) and between the control and dry AMD groups (*p* = 0.0024). There was no significant difference between the AMD subcohorts (*p* = 0.8826).

Complete vitreous separation in the posterior pole (stages 3 and 4) was not dependent on the number of intravitreal injections (IVIs), as shown in Table 3.

Analysis of BCVA in the AMD groups in terms of the PVD stage revealed significant variations in the wet AMD cohort (Table 4 (part a)). Nevertheless, when stages with and without complete separation of the vitreous in the macula were merged (1 + 2 versus 3 + 4), the difference in BCVA between these groups was not significant (Table 4 (part b)).

Retinal thickness was not influenced by the PVD stage in any zone (Table 5, Table 6 and Table 7). These findings were consistent across both AMD subgroups and the control group.

## 5. Discussion

The correlation between PVD and the evolution of AMD has been assessed in many studies employing standard field-of-view OCT equipment, typically providing central scans of 6 mm × 6 mm. To our knowledge, PVD has been analyzed with UWF–OCT in only two papers: one focused on healthy individuals [8] and one focused on highly myopic patients with retinoschisis [9]. Thus, our study is the first to use widefield OCT to analyze PVD in AMD patients.

UWF–OCT provides a better view of the peripheral vitreoretinal interface, increasing early-stage PVD diagnostic precision. We believe that findings regarding the prevalence and evolution of PVD in controls and AMD patients are more reliable when using UWF–OCT. Our study, which included an age-controlled analysis of the prevalence of PVD in AMD eyes versus controls, revealed a significantly greater frequency of PVD in patients with macular degeneration. Hence, the role of vitreomacular traction present during vitreous separation cannot be excluded as a contributing factor in AMD development. Nevertheless, there was no significant difference in the presence of PVD between dry and wet AMD subcohorts; thus, the simple mechanical aspect of vitreoretinal traction cannot be the only factor considered in the etiology of AMD subtypes. Moreover, it appears that separation of the vitreous from the macula does not correlate directly with a patient’s BCVA. No differences in BCVA between AMD patients with PVD stages 1 + 2 and those with stages 3 + 4 were observed.

All wet AMD patients in our study received intravitreal anti-VEGF treatment. Hence, the possible correlation between vitreoretinal adhesion and intravitreal treatment must also be addressed. Among patients who received IVIs, there was no significant difference in IVI number between patients with complete PVD separation at the macula (stages 3 and 4) and those with the vitreous still attached to the posterior pole (stages 1 and 2). Therefore, the vitreous detachment process is independent of the number of IVIs received. This finding is consistent with previous studies investigating IVIs and PVD frequency; Veloso et al. also found no relationship between PVD and the number of IVIs [10]. The same author reported release of the vitreous in just 30% of patients after IVI treatment for diabetic macular edema [11]. These findings are consistent with our analysis.

In the absence of UWF–OCT studies on AMD, we must refer to research performed using standard OCT devices. The results of the available studies are not unequivocal regarding the relationship between the vitreomacular interface and the presence of AMD. A population-based study by Gattoussi et al. indicated no association between AMD and vitreomacular adhesion (VMA) [12]. Conversely, a large meta-analysis revealed a greater incidence of VMA in wet AMD patients [13]. A recent study by Bakaliau et al. revealed less frequent transformation of dry AMD eyes than wet AMD eyes in patients with complete PVD [14]. Other authors have suggested the role of vitreoretinal traction in the development of exudative AMD [15,16,17]. Kang et al. hypothesized that VMA can stimulate inflammation, impair retinal oxygenation and VEGF production, and subsequently lead to the development of choroidal neovascularization [18].

Our analysis supports the involvement of the vitreomacular interface in the development of AMD. However, in contrast to some of the abovementioned studies, we did not find more frequent VMA in the wet AMD group or better BCVA in eyes with complete vitreous separation. Nevertheless, as we mentioned earlier, the early occurrence of PVD may add to the etiopathogenesis of AMD, not necessarily through simple mechanical traction. Stimulation of inflammation and impaired oxygenation can also be triggered by VMA, which occurs early in AMD development but does not prevent vitreous separation. Hence, we observe an already separated vitreous along with active biochemical degeneration pathways in developed AMD.

According to our study results, separation of the vitreous does not appear to significantly affect retinal thickness (Table 5, Table 6 and Table 7). Theoretically, stronger adhesion of the vitreous and subsequent possible traction should increase the retinal thickness; however, no such correlation was found in our study. This finding also supports the hypothesis that the relationship between the development of AMD and VMA cannot be strictly mechanical.

## 6. Study Limitations

This study has some limitations. First, this was a retrospective study, and the precise onset of PVD and AMD was not possible to determine, making it difficult to analyze the explored relationship in detail.

Second, the control and AMD groups demonstrated different mean ages. As it is difficult to compose an older group of subjects without signs of AMD, we had to accept a younger cohort as the control group. This approach required age correction during the statistical analysis.

Third, the development of diseases such as AMD is a complex and multifactorial process; hence, the relationship between AMD and PVD is just one factor in a wider process.

## 7. Conclusions

UWF–OCT testing of AMD patients provides reliable results regarding vitreoretinal interface involvement and retinal thickness. PVD is significantly more common in AMD patients than in controls, indicating its role in the pathogenesis of AMD development; nevertheless, exploration of this association requires further studies. BCVA in AMD patients is not directly related to the PVD stage.

## Figures and Tables

**Table 1:** Baseline characteristics of the study cohort by AMD presence and type (discrete variables).

Analyzed Trait	Controls	Wet AMD	Dry AMD	*p* Value
Number of participants, *n* (%)	53 (23.77)	87 (39.01)	83 (37.22)	
Number of eyes, n (%)	85 (25.68)	123 (37.16)	123 (37.16)	
Sex, n (%):				
- Female	26 (49.06)	58 (66.67)	60 (72.29)	0.0192
- Male	27 (50.94)	29 (33.33)	23 (27.71)	
Age (years)	68.02 +/− 8.78	74.54 +/− 6.87	74.43 +/− 5.78	<0.0001
Axial length [mm]	24.67 +/− 1.24	24.78 +/− 1.67	24.97 +/− 1.9	0.4174
BCVA (logMAR)	0.0	0.61 +/− 0.44	0.21 +/− 0.3	<0.0001
AMD stage, n (%):				
1	na	0 (0.00)	0 (0.00)	
2	na	0 (0.00)	21 (17.07)	
3	na	0 (0.00)	92 (74.80)	
4	na	123 (100)	10 (8.13)	

BCVA—best corrected visual acuity, AMD—age-related macular degeneration, n—number, na—not applicable.

**Table 2:** (**A**) Variations in PVD stages in the study and control groups controlled for age and sex (multivariate analysis). (**B**) Significance of pairwise comparisons between the study and control groups (*p* values) controlled for age and sex.

(A)				
Analyzed Trait	Controls	Wet AMD	Dry AMD	*p* Value
PVD stage, n (%):				
1a	15 (17.65)	2 (1.63)	0 (0.00)	=0.0006
1b	11 (12.94)	8 (6.50)	15 (12.20)	
2	12 (14.12)	21 (17.07)	15 (12.20)	
3	7 (8.23)	26 (21.14)	13 (10.57)	
4	40 (47.06)	66 (53.66)	80 (65.03)	
(**B**)				
	**Controls**	**Wet AMD**	**Dry AMD**	
Controls	X	0.0084	0.0006	
Wet AMD	0.0084	X	0.2180	
Dry AMD	0.0006	0.2180	X	

PVD—posterior vitreous detachment, AMD—age-related macular degeneration, n—number, AMD—age-related macular degeneration.

**Table 3:** Descriptive statistics for intravitreal injections in patients with wet AMD by PVD stage (n = 123 eyes).

PVD Stage	Number of IVI				*p* Value *
	M	SD	Me	Q_1_–Q_3_	
1 + 2	10.27	4.45	10.00	8.00–12.00	=0.2634
3 + 4	9.42	5.71	8.00	5.00–13.00	

M—mean; SD—standard deviation; Me—median; Q—quartiles. * Controlled for sex and age.

**Table 4:** (**a**). Descriptive statistics for BCVA (logMAR) by type of AMD (n = 246 eyes) (multivariate analysis). (**b**) Differences in BCVA values between AMD subcohorts in reference to complete separation of the vitreous in the posterior pole: stage 1 + 2 versus 3 + 4 (n = 246 eyes) (multivariate analysis).

(a)						
BCVA logMAR	PVD Stage	Statistical Parameter *				*p* Value **
		M	SD	Me	Q_1_–Q_3_	
Wet AMD	1a	0.50	0.14	0.50	0.40–0.60	=0.0078
	1b	1.09	0.64	0.85	0.65–1.50	
	2	0.51	0.34	0.40	0.30–0.70	
	3	0.61	0.49	0.40	0.30–0.70	
	4	0.59	0.41	0.50	0.30–0.70	
Dry AMD	1a	n/d	n/d	n/d	n/d	=0.1925
	1b	0.31	0.33	0.25	0.05–0.50	
	2	0.17	0.19	0.10	0.10–0.20	
	3	0.22	0.39	0.10	0.00–0.25	
	4	0.20	0.30	0.10	0.00–0.20	
**(b)**						
**BCVAlog** **MAR**	**PVD Stage**	Statistical Parameter *				*p* Value **
		**M**	**SD**	**Me**	**Q_1_–Q_3_**	
Wet AMD	1 + 2	0.51	0.36	0.40	0.30–0.70	=0.1844
	3 + 4	0.65	0.46	0.60	0.30–0.70	
Dry AMD	1 + 2	0.23	0.32	0.10	0.00–0.30	=0.8190
	3 + 4	0.21	0.30	0.10	0.10–0.20	

* Statistical measures used: M—mean; SD—standard deviation; Me—median; Q—quartiles; BCVA—best corrected visual acuity. ** Controlled for sex and age.

**Table 5:** Descriptive statistics for central retinal thickness (µm) by AMD type and PVD stage (n = 331 eyes) (multivariate analysis).

Central Retinal Thickness (µm)	PVD Stage	Statistical Parameter *				*p* Value **
		M	SD	Me	Q_1_–Q_3_	
Controls	1a	341.47	16.26	339.00	328.00–358.00	=0.3353
	1b	331.64	14.14	334.00	323.00–341.00	
	2	344.58	20.60	350.00	333.00–357.50	
	3	323.43	16.29	332.00	305.00–334.00	
	4	331.77	24.72	332.00	322.00–346.50	
Wet AMD	1a	306.50	43.13	306.50	276.00–337.00	=0.2532
	1b	346.50	64.06	331.50	294.50–399.50	
	2	331.33	48.08	320.00	303.00–340.00	
	3	343.04	62.99	330.00	305.00–368.00	
	4	319.24	50.82	310.00	285.00–351.00	
Dry AMD	1a	n/d	n/d	n/d	n/d	=0.0534
	1b	320.73	27.16	321.00	302.00–351.00	
	2	318.47	24.79	325.00	295.00–341.00	
	3	322.46	21.52	325.00	305.00–345.00	
	4	329.51	19.67	330.00	317.00–342.50	

* Statistical measures used: M—mean; SD—standard deviation; Me—median; Q—quartiles. ** Controlled for sex and age.

**Table 6:** Descriptive statistics for perifoveal retinal thickness (µm) by AMD type and PVD stage (n = 331 eyes) (multivariate analysis).

Perifoveal Retinal Thickness (µm)	PVD Stage	Statistical Parameter *				*p* Value **
		M	SD	Me	Q_1_–Q_3_	
Controls	1a	286.76	13.21	287.56	274.87–299.12	=0.7389
	1b	289.12	9.51	289.44	281.69–296.62	
	2	291.05	13.72	289.62	281.25–297.97	
	3	278.33	12.51	279.00	263.75–219.19	
	4	284.18	15.98	281.87	275.87–296.69	
Wet AMD	1a	266.97	40.88	266.97	238.06–295.87	=0.1323
	1b	267.60	40.71	269.94	231.87–283.56	
	2	278.04	18.45	281.06	263.06–288.94	
	3	289.16	36.17	288.84	257.75–302.37	
	4	275.59	25.16	273.37	260.56–295.25	
Dry AMD	1a	na	na	na	na	=0.4205
	1b	282.35	16.68	280.69	270.06–299.00	
	2	281.27	15.75	279.62	273.81–291.06	
	3	285.55	15.62	282.50	272.75–289.00	
	4	286.47	15.28	284.16	276.31–295.47	

na—not applicable. * Statistical measures used: M—mean; SD—standard deviation; Me—median; Q—quartiles. ** Controlled for sex and age.

**Table 7:** Descriptive statistics for peripheral retinal thickness (µm) by AMD type and PVD stage (n = 331 eyes) (multivariate analysis).

Peripheral Retinal Thickness (µm)	PVD Stage	Statistical Parameter *				*p* Value **
		M	SD	Me	Q_1_–Q_3_	
Controls	1a	220.91	8.78	223.75	211.87–226.12	=0.1902
	1b	225.51	14.66	228.87	212.12–235.87	
	2	225.13	13.11	226.62	213.50–234.12	
	3	209.73	12.12	208.00	199.75–216.12	
	4	218.70	11.81	220.94	206.87–225.87	
Wet AMD	1a	217.81	32.97	217.81	194.50–241.12	=0.2912
	1b	222.47	16.88	225.44	209.06–235.94	
	2	219.17	10.61	218.00	212.87–227.50	
	3	224.80	14.11	228.25	214.12–234.37	
	4	218.62	15.43	219.50	206.00–229.25	
Dry AMD	1a	na	na	na	na	=0.4011
	1b	217.07	14.44	216.12	209.75–222.50	
	2	218.67	13.16	218.12	208.75–225.00	
	3	217.56	12.93	218.37	206.62–224.37	
	4	224.43	12.03	226.37	213.94–233.31	

na—not applicable. * Statistical measures used: M—mean; SD—standard deviation; Me—median; Q—quartiles. ** Controlled for sex and age.

## Data Availability

Additional resource data are available from the corresponding author upon request.
